# Decolonising violence against women research: a study design for co-developing violence prevention interventions with communities in low and middle income countries (LMICs)

**DOI:** 10.1186/s12889-021-11172-2

**Published:** 2021-06-15

**Authors:** Jenevieve Mannell, Safua Akeli Amaama, Ramona Boodoosingh, Laura Brown, Maria Calderon, Esther Cowley-Malcolm, Hattie Lowe, Angélica Motta, Geordan Shannon, Helen Tanielu, Carla Cortez Vergara

**Affiliations:** 1 Institute for Global Health, University College London, London, UK; 2 Te Papa Museum, Wellington, New Zealand; 3 National University of Samoa, Apia, Samoa; 4 Hampi Consultores en Salud, Lima, Peru; 5 Department of Anthropology, San Marcos University, San Marcos, Peru; 6 Cayetano Heredia University, Lima, Peru

**Keywords:** Violence against women; indigenous perspectives, Southern epistemologies, Co-design, Participatory research, Samoa, Peru

## Abstract

**Background:**

There has been substantial progress in research on preventing violence against women and girls (VAWG) in the last 20 years. While the evidence suggests the potential of well-designed curriculum-based interventions that target known risk factors of violence at the community level, this has certain limitations for working in partnership with communities in low- and middle-income (LMIC) countries, particularly when it comes to addressing the power dynamics embedded within north-south research relationships.

**Methods:**

As an alternative approach, we outline the study design for the EVE Project: a formative research project implemented in partnership with community-based researchers in Samoa and Amantaní (Peru) using a participatory co-design approach to VAWG prevention research. We detail the methods we will use to overcome the power dynamics that have been historically embedded in Western research practices, including: collaboratively defining and agreeing research guidelines before the start of the project, co-creating theories of change with community stakeholders, identifying local understandings of violence to inform the selection and measurement of potential outcomes, and co-designing VAWG prevention interventions with communities.

**Discussion:**

Indigenous knowledge and ways of thinking have often been undermined historically by Western research practices, contributing to repeated calls for better recognition of Southern epistemologies. The EVE Project design outlines our collective thinking on how to address this gap and to further VAWG prevention through the meaningful participation of communities affected by violence in the research and design of their own interventions. We also discuss the significant impact of the COVID-19 pandemic on the project in ways that have both disrupted and expanded the potential for a better transfer of power to the communities involved. This article offers specific strategies for integrating Southern epistemologies into VAWG research practices in four domains: ethics, theories of change, measurement, and intervention design. Our aim is to create new spaces for engagement between indigenous ways of thinking and the evidence that has been established from the past two decades of VAWG prevention research and practice.

## Background

Over the past 20 years, there has been substantial progress made in research on how to prevent violence against women and girls (VAWG) in low and middle income countries (LMICs) [1, 2]. The extent of the problem and risk factors for VAWG have been well documented [3–5], as have the serious mental and physical health consequences of violence for women’s lives [6–8]. In order to address violence and its consequences in LMICs, available evidence points to the potential of community-based interventions that use group training and community mobilisation techniques to prevent VAWG [9]. A handful of cluster randomised trials of curriculum-based community mobilisation interventions have equally demonstrated that reducing the prevalence of violence in relatively short timeframes is indeed possible [10–12], and recent evidence from the global programme on “What Works to Prevent Violence against Women and Girls” emphasises that carefully planned interventions adapted for local contexts with a clear theory of change can achieve positive outcomes [13].

While well-designed interventions that target risk factors of violence is a useful starting point for thinking about how to design successful VAWG prevention interventions, the adaptation of predefined curricula in different settings around the world raises concerns around power and privilege when working with communities in LMICs with a colonial history. The social and structural factors that contribute to high levels of VAWG, including gender inequalities, extreme poverty and social marginalisation, are often magnified for communities with a legacy of colonialism [14]. For many postcolonial scholars, the violence of colonialism is intimately connected to the high rates of VAWG currently experienced in many LMICs and in aboriginal communities globally [15]. In much the same way, colonialism and new forms of imperialism are often blamed for undermining Southern practices and epistemologies, or ‘ways of knowing’, as part of research practices [16]. This has led to calls for ‘epistemic justice’ through a recognition of Southern epistemologies [17], e.g. theories of how knowledge is obtained, justified and reproduced that are historically aligned with the beliefs and practices of pre-colonial societies. In order to integrate Southern epistemologies into VAWG prevention interventions, the structural inequalities that have marginalised indigenous forms of knowledge need to first be addressed.

Towards this goal, we outline a study design for a novel participatory approach to involving communities affected by violence and with a history of colonialism in the design of their own VAWG prevention interventions. We use a place-based definition of community in this article, whereby communities are geographically and conceptually linked to a location or physical place. We argue that the participation of communities is necessary to address the limitations of current approaches to VAWG prevention research and to reverse the colonial practices that have contributed to the exclusion of a Southern perspective in intervention design and evaluation research. We discuss how the current context of the COVID-19 pandemic has also provided an opportunity for rethinking research relationships and giving more control over research ideas to local communities. As a means of bringing Southern epistemologies into VAWG prevention research, we have prioritised four research-related domains and outline a participatory study to address these domains as part of a strategy for decolonising our own research practices.

### Decolonising VAWG research using participatory approaches to intervention design

Throughout the colonial period lasting from the 15th to the late twentieth century, research was often used as a means of re-affirming and consolidating the colonial project and the dominance of the coloniser over the colonised [18, 19]. For example, Turball outlines how Australian aboriginal remains were removed from ancient burial grounds as part of the scientific study of racial difference, and the justification of the colonial belief in the inferiority of aboriginal bodies [20]. Others argue that the extractive tendencies of Euro-Western research practices did not end with the colonial period, and that new forms of imperialism and post-colonial relationships between the researcher and the researched still persist in today’s research practices [21–23]. For instance, Latin American scholars have pointed to the ways in which the legacy of colonialism still configures social life in the region through current economic arrangements and political systems [24].

To address the legacy of colonial histories and new imperial relationships, scholars in Australia, New Zealand and Canada in particular have drawn attention to the importance of an anti-colonial approach to research [25–28]. These scholars point to the essential need to conduct research about indigenous issues in meaningful collaboration with communities and ensure that an indigenous worldview underpins research with direct benefits to the communities involved [16, 29]. Pacific researchers in particular have pointed to a unique ontology of research from the perspective of Pacific indigenous communities founded in relationality rather than individualistic values [28, 30]. Despite growing scholarship that adopts this more critical approach to research practices with indigenous populations [31, 32], an anti-colonial perspective has rarely been applied to research on VAWG prevention. As a potential explanation, scholars point to the hard-won assumption by feminist researchers that VAWG is directly caused by patriarchy and gender inequalities, which can undermine attention to indigenous explanations for VAWG as rooted in structural forms of violence that are also experienced by men [27]. Others have pointed to how the current emphasis on gender inequality as a key driver of violence in Northern scholarship may obscure the way that gender has been historically constituted [33, 34], and how this has been often been accomplished specifically through acts of VAWG (e.g. the rape of indigenous women and its role in reconfiguring sexuality, inheritance and notions of family) [35].

The aim of this article is not to provide a critique of research practices in VAWG research, which has been provided by others [27, 36]. Instead, we reflect on how participatory approaches can be used to create spaces that support dialogue between best practices in VAWG research and Southern epistemologies. This is consistent with what has been described as working as an ‘ally’ with marginalised populations to do their own research, or knowledge-sharing as part of a process of co-production [27, 37]. It also responds to calls for a solidarity-based epistemology characterised by horizontal formations of knowledge and mutual learning [38]. The participation of communities in research design, data collection and analysis is well recognised for its advantages in addressing the power dynamics that underpin research engagements [39], and is often seen as aligned with indigenous approaches to research in the Pacific and Latin American traditions [31, 40]. In this way, participatory research approaches offer a means of surfacing Southern epistemologies by drawing attention to the ‘epistemic privilege’ of mainstream research approaches, exposing the power dynamics embedded in knowledge production, and providing a space for open discussion [41].

This paper is organised around four domains of VAWG prevention research design: (1) ethical guidelines, (2) theories of change, (3) outcome measurement, and (4) intervention development. We discuss each of these domains in turn, briefly summarising the relevant literature within VAWG research and then discussing how we have used participatory methods to bring a Southern perspective into dialogue with the literature as part of the EVE Project (Evidence for Violence prevention in the Extreme).

### The EVE project

The EVE Project is a mixed methods participatory project funded by UK Research and Innovation, which started in March 2020. The aim of this research project is to establish an evidence-base for how to prevent VAWG in high-prevalence settings globally (defined as settings with a prevalence of past year physical and/or sexual violence greater than the global median of 11.4%). The project has four main objectives, which are aligned with the four domains of VAWG prevention research design.

## Methods/ design

The EVE Project includes two case studies of community-based research with communities to develop VAWG prevention interventions in Amantaní, an island located in Lake Titicaca of the Peruvian Andes, and Samoa, a group of islands in the Polynesian region of the Pacific. Both of these communities are situated in post-colonial contexts: which we have defined as previously colonised spaces, including either nations or populations within countries, characterised by new forms of imperialism [21, 42, 43].

### Research settings

#### Amantaní, Peru

The island of Amantaní is inhabited primarily by a Quechua-speaking indigenous population. This population has reported extremely high rates of partner violence: according to the Demographic and Family Health Survey (ENDES) from 2019, 62.5% of women between the ages of 15 and 49 of native origin, including women self-identified as Quechua, experienced some type of violence exerted by their partner during their lives, markedly higher than the national prevalence (57.7%) [44]. The most common form of violence against women of native origin is psychological and/or verbal (57.5%). In addition, Amantaní is located in the broader region of Puno, which has amongst the highest prevalence rates of intimate partner violence in the country. 63.4% of women living in Puno report ever experiencing violence from a husband or partner, with 37.3% experiencing some form of intimate partner violence within the last year [44]. These high rates reflect socio-cultural views of violence as normal in women’s lives, the way in which Latin American history has reinforced community identities in Andean communities [45], and broader indigenous experiences of discrimination [46].

#### The Independent State of Samoa

The Pacific islands is the region with the highest prevalence of VAWG in the world: 68% of women will experience physical or sexual violence in their lifetime [6]. In Samoa, a recent report from the Office of the Ombudsman reported that 86% of women currently experience physical violence from an intimate partner including kicking, punching and slapping [47]. Samoa has a complex society dating back 3000 years where family (*āiga*) and village (*nu’u*) structures are at the heart of social, political and economic organisation [48]. Since the arrival of Christian missionaries in the 1830s, the church also plays a significant role in cultural life and the social organisation of Samoan society [49]. The effects of colonising research practices on understanding these social structures are evident in long-standing debates among anthropologists that represent Samoan society as either inherently violent [50], or a unique example of a culture without conflict [51]. These anthropological accounts of violence in Samoa have been widely critiqued by postcolonial scholars who have highlighted the consequences of misinterpreting the meanings of Samoan cultural traditions through outsider research practices [52, 53].

### Community engagement and selection

In both Peru and Samoa, as part of the EVE Project, community-based researchers (CBRs) have been engaged through organisations with a history of working with local communities. Our local Peruvian organisation, Hampi Consultores en salud (https://hampiconsultores.com/), has previously conducted population-based surveys on health and violence with Quechua communities and has significant local contacts and resource networks in the area [54]. Our Samoan partner organisation, the Samoa Victim Support Group (SVSG) (http://www.samoavictimsupport.org/), is a Samoan non-governmental organisation established in 2005 to provide an integrated, personalised, professional service to survivors of domestic violence. SVSG provides training and support for village representatives across Samoa to respond to domestic violence and sexual abuse cases.

The selection of communities in both settings will take into consideration the diversity of the communities, reflecting on dynamics such as community leadership and structure, population, accessibility, and proximity to urban areas. Community selection will also be guided by practical considerations including existing relationships and the level of trust between the implementing organisation and local leaders.

### CBR selection and training

#### Community-based researchers

**Fig. 1:**
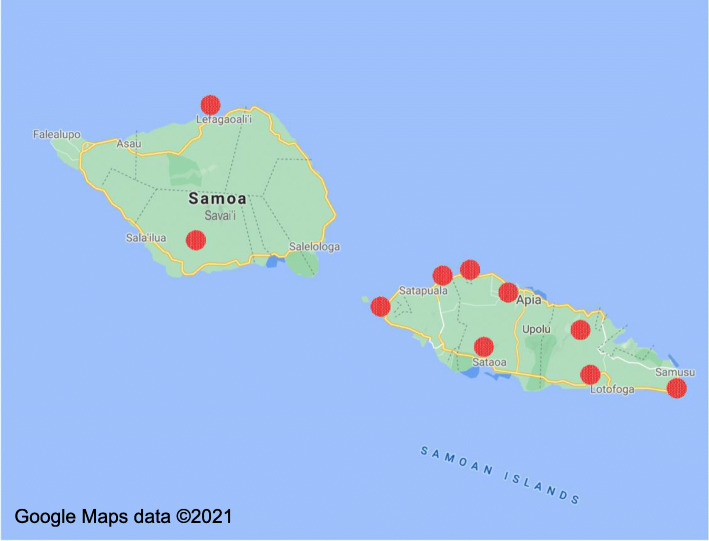
Samoan communities (villages) [87]

The community-based researchers (CBRs) hired as part of the EVE Project will be responsible for making the majority of decisions about how the project will be implemented. In Samoa, 20 CBRs from 10 villages will be purposively selected by SVSG from their existing network of over 1000 community representatives across the country (see Fig. 1). Selection of CBRs will be based on criteria including: gender balance (one man and one woman from each community), technical knowledge (e.g. ability to use mobile technology), and status within the community (necessary to facilitate community conversations). A mentor will also be selected by the CBRs from their community to ensure that the knowledge of community elders is integrated into the project design from its inception, and that local customs around appropriate communication are taken into consideration [48].

**Fig. 2:**
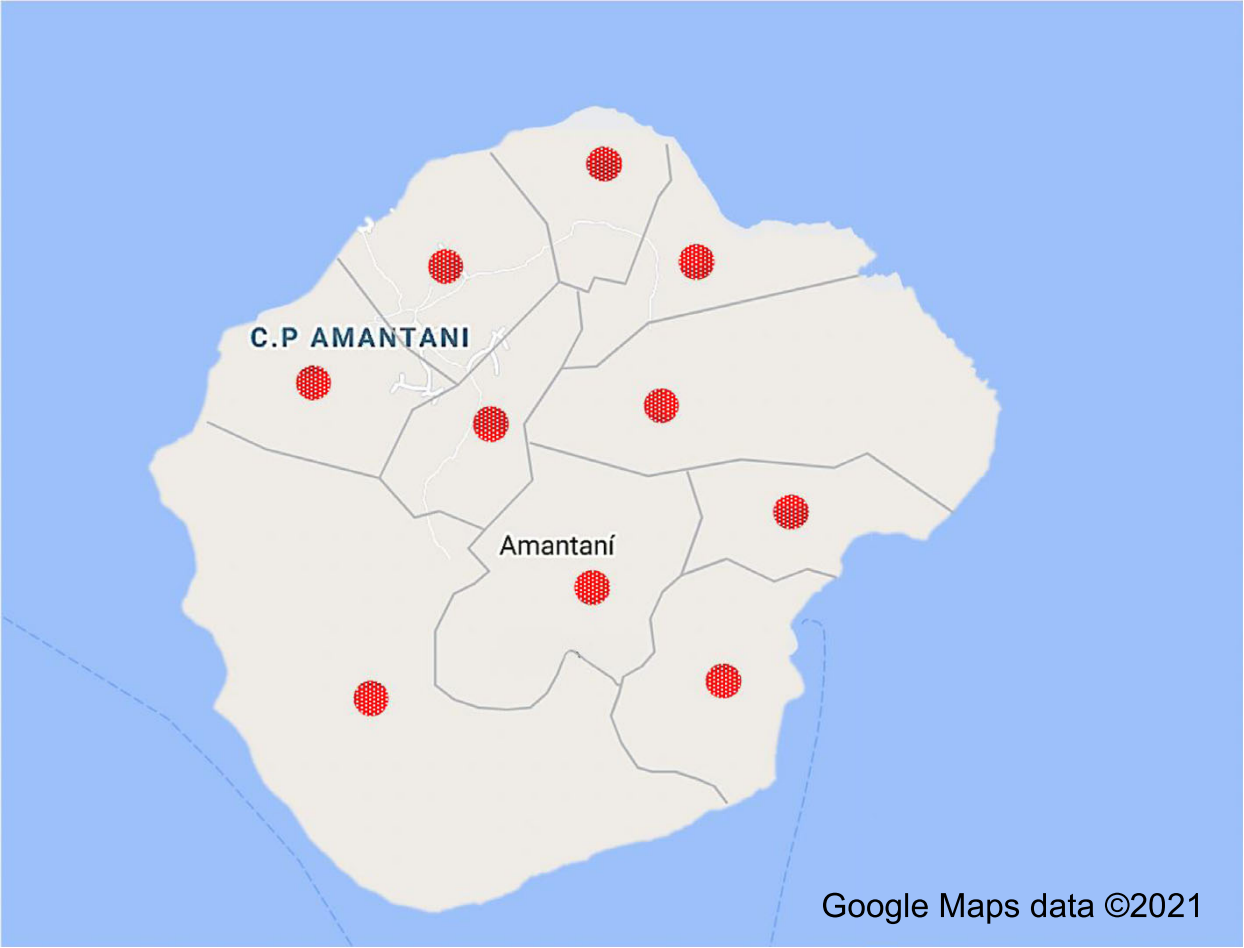
Amantaní communities [88]

Hampi does not have an existing network of village representatives to draw on, and CBRs will therefore be identified using snowballing techniques and one-to-one conversations with community members and local leaders. A total of 10 CBRs will be identified; one from each of Amantaní’s 10 districts (see Fig. 2). Selection of the CBRs will be based on geographic diversity, and the ability of the CBR to communicate via technology (e.g. mobile phone). Given low literacy rates among women on the island, this will not be a requirement for participation of the CBRs and all activities will be adapted to make their full participation possible. The Amantaní CBRs will all be women to address potential concerns around power dynamics undermining women’s perspectives in mixed groups.

In both Peru and Samoa, CBRs will be involved in 170 h of structured participatory workshops as part of the project, provided by SVSG and Hampi. These workshops will provide a means of discussing the ideas and assumptions of VAWG scholars about the drivers of violence and strategies for its prevention, and training CBRs in research techniques including semi-structured interviews and thematic analysis. Workshop activities will encourage input by the CBRs into the research process (e.g. by altering topic guide questions and formats, collaboratively discussing participant selection, discussing when privacy may be necessary and how to achieve it, etc.). The research process itself will be done iteratively with the potential for CBRs to make significant changes to the types of data collected, how to analyse these data, and themes to include in the study’s results.

The CBRs will also provide their own data as part of the project through three interviews at the beginning, middle and end. These interviews will be semi-structured to include topics such as their individual experiences to date, any challenges raised, and their own thoughts and ideas about how violence could be prevented in their community. This will help to ensure that every CBR is able to input their own ideas into the project design and not only those who are most outspoken during workshops. It also provides a means of understanding how the project is progressing and the reasons behind challenges or changes that need to be made.

#### Community participants

The CBRs in each setting will select participants from their communities to participate in the study. During a training workshop on research techniques, CBRs will take part in an activity to discuss who they might select. CBRs will be encouraged to think creatively, considering all community members that may have knowledge on the problem of VAWG, rather than only selecting victims or survivors of violence and community members in positions of power. In Samoa, men will be encouraged to interview men and women to interview women to be considerate of local gender norms and to encourage open and honest dialogue.

### Decolonising research about violence against women and girls

The four domains of VAWG research targeted as part of the EVE Project correspond with the project’s objectives and timeline. Each domain represents an independent phase of the project: Phase 1 ‘Developing Ethical Guidelines’; Phase 2 ‘Theory of Change’; Phase 3 ‘Outcome Measurement’; and Phase 4: ‘Intervention development’. We have integrated a space for interaction between the UK-based research team and CBRs, and between the CBRs and the communities they represent as part of each component, as outlined in Table 1.

**Table 1:** The EVE Project objectives and design components

Objectives	Design Components	Methods
To co-create ethical guidelines for violence prevention research and intervention in collaboration with high-prevalence communities	1. Developing ethical guidelines	Semi-standardised interviews Participatory hermeneutic analysis
To establish the causal mechanisms for how community participation prevents VAWG in high-prevalence settings	2. Developing theories of change	Stories of change as case studies Collaborative thematic analysis
To develop, validate and feasibility-test new tools for assessing VAWG prevalence in high-prevalence settings	3. Outcome measurement	Participatory listing/ ranking exercises Focus group discussions
To co-create an intervention in collaboration with high-prevalence communities	4. Participatory Community-led Intervention Development (PCID)	Participatory action research workshops and activity testing

#### Phase 1: developing ethical guidelines

In 2001, researchers belonging to the International Research Network on Violence Against Women (IRNVAW) developed a set of ethical guidelines for VAWG researchers that were later published by the World Health Organisation (WHO) and widely recognised as ethical standards for the field [55]. Several scholars have since discussed how the guidelines need to be adapted for particular settings [56–59]. The adaptations of the WHO guidelines for several purposes and contexts points to the need for a more *situated* approach to ethical engagement [57, 60]. Towards this goal, the project is designed to use semi-standardised interviews conducted by each CBR with 3–5 community participants to identify the moral decision-making process [61] that community members use to respond to cases of violence. Semi-standardised interviews are a social psychological tool used to investigate subjective theories during the interview process [62]. This is done by asking questions that are designed to elicit both explicit and implicit knowledge on a topic, for example, direct questions about how community members are responding to VAWG and more controversial questions around what participants thought of ideas that are widely accepted by VAWG researchers (e.g. that childhood experiences of violence are a risk factor for experiencing and perpetrating violence later in life) [5].

The CBRs will then participate in a workshop where they will use the data from the interviews to develop a unique set of ethical guidelines for each setting. The aim of the workshop is to ensure that CBRs’ reflections on the moral reasoning of participants provides the basis for the identification of themes from the data. We have developed a unique approach for accomplishing this drawing on a hermeneutic phenomenological analysis of ethical decision-making processes first described by Lindseth and Norberg [63], and adapting it for use as a participatory method as outlined in Table 2.

**Table 2:** Participatory hermeneutic analysis for the development of ethical guidelines

Hermeneutic Phenomenological Analysis of ethical decision-making [63]	Participatory hermeneutic analysis for the EVE Project
**Stage 1**: Developing a naïve interpretation of the meaning of the topic under investigation	Semi-standardised interviews about the meaning of VAWG with community participants, conducted by CBRs; Drawing from the interviews and in collaboration with the research team, CBRs develop a set of guiding phrases for how people should respond to a woman in the community experiencing violence;
**Stage 2**: Identifying themes and sub-themes, and back-checking these against the naïve interpretation	CBRs identify themes arising from the interviews about decisions around responding to women experiencing violence; Grouping these themes into organising categories (higher order themes);
**Stage 3**: Validate themes against stories of lived experience that tell us about the essence of the topic under investigation	CBRs validate the guiding phrases about how community members should respond to violence against the themes and categories; Further validation provided through comparison with local myths and stories.

We will adapt this process to draw directly on the CBRs’ interpretations of the interviews by leading them as a group through a simplified version of these three stages, which results in a set of guiding phrases/ guidelines for how communities (and the research team) should respond to women experiencing violence. This then provides a reference point for developing practical strategies for implementing WHO guidelines, developing new ethical guidelines where needed, and making informed decisions about adaptations to project activities.

#### Phase 2: Theories of Change

The few models that exist for reducing VAWG primarily draw on the behavioural sciences. These models have been widely used to develop strategies for addressing different risk factors for VAWG, including toxic masculinity, social norms of gender and violence, conflict within family and intimate relationships, and harmful drinking behaviours [64–66]. As part of a decolonising approach, local understandings of violence and community perceptions of its solutions should also inform our theory of change for the EVE Project [67].

To deliver this, we will develop a theory of change in partnership with CBRs using data they have collected from their own communities. This will involve using the semi-standardised interviews collected by CBRs in Phase 1 to collaboratively develop a ‘story’ of violence prevention for each individual community, which can then be used as a case study for analysis. These stories of violence will be developed through a series of participatory activities designed to help CBRs create a narrative from the interviews they have collected. The participatory activities will help facilitate the development of characters for each community’s story, a narrative sequence of events, and a main objective or purpose for the story. The final case studies may discuss either positive or negative examples of violence prevention, and will explicitly talk about how community involvement contributes to either increased or decreased violence in the community. Once developed, the case studies will be collaboratively analysed by the CBRs to identify causal mechanisms of violence prevention evidenced in the community case studies. The co-produced theory of how community involvement contributes to violence prevention will then be presented by the CBRs back to their communities for community discussion and input before being finalised.

The use of storytelling to develop case studies in this way is underpinned methodologically by the importance of stories in both generating spaces for social change and accounting for changes that have taken place. Samoans have rich oral traditions of stories and myths, which have been used by others to understand the rich cultural history of Samoan society [68, 69]. This provides a basis for using stories to understanding local meanings of violence prevention in this study design.

#### Phase 3: outcome measurement

In the vast majority of VAWG research, the primary outcome of interest is the reduction of VAWG. However, what constitutes violence and what level of reduction is meaningful in the lives of women is highly subjective and context-specific. The vast majority of VAWG survey tools draw on the Conflict Tactics Scale (CTS) [70], which asks about specific acts of physical violence (e.g. being hit, beaten, slapped, kicked, etc.), psychological abuse, and sexual coercion. While asking about specific acts of violence has provided an opportunity for understanding the extent of violence when individuals may not themselves consider certain actions violent or harmful, the list of actions included in the majority of survey tools fails to account for acts of VAWG that fall outside of the sphere of either intimate partner violence or non-partner rape, such as acid attacks, honour killings, and sexual slavery [71–73]. The CTS and its variations have also been critiqued for not adequately capturing how gender inequalities and contexts of coercive control influence violent behaviours [74, 75]. A broader understanding of the types of violence women may experience may be needed to capture the full extent of the problem in different contexts.

In the EVE Project, we address this by first asking what types of violence are recognised as important to a diverse range of community members during the interviews conducted by CBRs. CBRs will be asked to probe for types of violence that may not be immediately recognised as violence, but that do cause harm to another individual, e.g. economic violence, controlling behaviours, honour-related violence, modern slavery. During the analysis workshop, we will ask CBRs to create a list of types of violence and to use the data to rank the importance of these types according to the importance they hold in women’s lives in each setting. This preliminary conceptualisation of VAWG will then be used as a basis for a series of 4–5 focus groups with women in communities about how the different types of violence impact their everyday lives and what a meaningful reduction in the violence would look like, exploring dimensions of type, frequency, and severity. The findings of these two data collection approaches will provide a basis for selecting and adapting relevant survey tools. The final survey tool will then be further adapted to local epistemologies using cognitive interview techniques [76].

#### Phase 4: participatory community-led intervention development (PCID)

The EVE Project will use a Participatory Community-led Intervention Development (PCID) approach first developed as part of the Gender-based violence prevention in the Amazon of Peru (GAP) Project [77]. PCID draws on well-recognised components of participatory action research (PAR) including: the participation of targeted groups in the research process to answer the questions they themselves define, a cyclical process of data collection and analysis, and conceptual attention to addressing structural forms of violence through asking critically-informed questions about the problem and proposed solutions (in this case, VAWG) [78, 79]. Similar to PAR, the PCID approach encourages participants to develop understandings of the inequalities that guide their behaviours and develop critical consciousness or *conscientização* [80] (e.g. about the structural reasons for a high prevalence of violence in their communities). This approach is aligned with the Latin American educationalist Paulo Friere, whose particular pedagogy involves engaging participants in asking critical questions about their lives and experiences rather than ‘teaching’ participants [81]. In the PCID approach, critical consciousness is achieved through engaging participants in identifying the reasons behind VAWG in their communities and designing an intervention to address it as part of the research/action cycle.

**Fig. 3:**
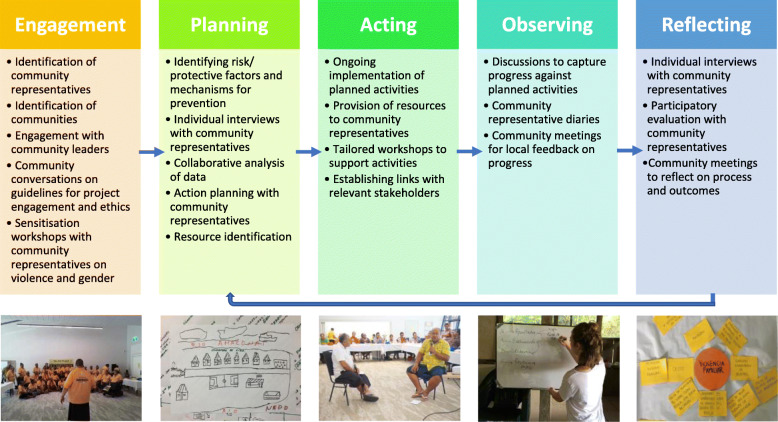
Participatory Community-led Intervention Development (PCID) approach for preventing violence against women

In practice, the PCID approach uses a combination of concept mapping, project management techniques, role play, and participatory evaluation activities with CBRs at different time points, to ensure a shared understanding of relevant concepts and to develop specific VAWG prevention intervention activities for communities as described in Fig. 3. The approach draws on the PAR cycle stages of planning, acting, observing and reflecting, which helps to ensure an iterative intervention design and long-term sustainability with minimal external input [77].

### Adapting the EVE project for COVID-19

The EVE Project in both Amantaní and Samoa will need to be adapted given restrictions in place surrounding the COVID-19 pandemic. The evolving global situation will have a differential impact on the two countries, and adaptations will need to vary across settings. Strict international travel restrictions were implemented in Samoa from as early as January 2020. Since then there have been only three confirmed COVID-19 cases in the country [82]. Whilst international borders remain closed, movement within Samoa is permitted as usual. In Peru, the first confirmed cases of COVID-19 were in March 2020. This was promptly followed by a strict national lockdown and a ban on international and domestic travel. At the time of writing, Peru was preparing for a possible third wave of the pandemic with additional tightening of restrictions and very limited mobility within and in and out of the country. The pandemic will impact the activities that can take place.

For example, to develop local ethical guidelines for the projects in both Amantaní and Samoa, the plan is to conduct a series of large community meetings at the beginning of the project to discuss and generate ideas. If this is not possible due to restrictions on large gatherings, ethical guidelines will be developed as part of a longer, more iterative process. CBRs will conduct interviews in their communities and work with the local organisations and the research teams in small groups to collaboratively develop these ethical approaches throughout the project. Moreover, training workshops for CBRs in Samoa will need to be delivered by the local partner organisation with a staff member at the National University of Samoa (NUS) acting as facilitator, instead of the UK research team as originally planned. As a respected local organisation, this will provide an opportunity for SVSG to foster a sense of community and togetherness during the training, which may not otherwise have been possible. This adaptation in particular will contribute towards increasing community involvement and ownership of the project; an overarching objective of the EVE Project.

As a result of restricted travel across Peru, the Peruvian team will conduct a remote health systems assessment as a method of gaining knowledge around the structures in place in Amantaní and Puno (the broader region) before community-based activities can begin. The exploration of how local governance works with regard to VAWG and what services are available to support victims and survivors will help to establish networks within the local area and lay the foundations for the project in Amantaní. Following this, the team will use a small number of local contacts in Amantaní to recruit 10 women as CBRs to begin collecting artefacts relevant to the project. This activity can be done remotely through the use of smartphones to capture images and provide a platform for the sharing of stories. This process will help to gain a better understanding of the local context, whilst also building relationships within the communities for the next phase of the project. This will enable the research process to be much more iterative and flexible, providing greater space for CBRs to contribute to the methodologies involved in the next phase. This is a necessity when working towards a decolonised approach to VAWG research; ensuring that it is informed by local constructions of knowledge and meaning.

## Discussion

This EVE Project study design described in this article reflects our collective thinking about how to decolonise our own research practices in VAWG research. This is an iterative process rather than a clearly defined prescriptive procedure. Trying to address power differentials that are embedded in research practices is a constant struggle between reflection, and trial and error. Our study design describes how we have brought social theory and participatory approaches into our reflections about who we are as researchers and the standpoint we take to VAWG prevention and response [21, 22].

We hope that this makes a welcome contribution to the field of VAWG research as part of an ongoing discussion with researchers, practitioners and activists about how we can better account for and recognise Southern epistemologies. The need for community participation to be an integral part of VAWG interventions is widely recognised [83], and the involvement of violence survivors and their communities as stakeholders and partners in research has been adopted as best practice [84]. However, survivors, perpetrators, and the communities they live in, are still rarely involved in the research process itself. They may be considered valuable research participants, but are rarely thought of as potential researchers. As we have argued throughout this article, acknowledging Southern epistemologies in VAWG research will require this integration of the people experiencing the violence into the research process.

The experience of COVID-19 and its impacts on international research relationships has brought the need to reconsider our research practices. The pandemic has demonstrated a clear need to be adaptable and constantly reflective about the ethical challenges and power structures that may be impacted, particularly with research on sensitive topics [85]. For the EVE Project, the COVID-19 pandemic has forced us to adjust our activities and give far more control over the research design to local partner organisations. We feel strongly that this will be beneficial to the outcomes of the project by making it more context-specific, more localised, and more grounded in Southern epistemologies. This equally provides an important lesson about the need to fundamentally shift the institutional structures that underpin global health research for the longer term [86].

A more critical perspective on decolonising mainstream research methodologies may argue that research frameworks and tools should ideally be developed entirely from the ground up with local communities driving the process according to their own needs [26]. In contrast to this, we have instead chosen a pragmatic approach that tries to establish a dialogue between Western and non-Western epistemologies, while constantly engaging in reflection about how to subvert the power dynamics this entails. This decision has its limitations, but we are optimistic about the possibilities it holds for drawing into question some of the widely held assumptions of VAWG research, while also recognising the decades of both Northern and Southern activism that have gone into shaping the field of VAWG research and practice over the past 20 years.

## Data Availability

Not applicable.

## Abbreviations

CBRs: Community-based researchers
CTS: Conflict tactics scale
EVE project: Evidence for Violence prevention in the Extreme
GAP project: Gender-based violence prevention in the Amazon of Peru
IRNVAW: International Research Network on Violence Against Women
LMICs: Low- and middle-income countries
NUS: National University of Samoa
PAR: Participatory action research
PCID: Participatory community-led intervention development
SVSG: Samoa Victim Support Group
VAWG: Violence against women and girls
WHO: World Health Organization
